# The Effect of Cryogenic Treatment and Tempering Duration on the Microstructure and Mechanical Properties of Martensitic Stainless Steel 13Cr-2Ni-2Mo

**DOI:** 10.3390/ma18081784

**Published:** 2025-04-14

**Authors:** Muhammad R. R. Fatih, Hou-Jen Chen, Hsin-Chih Lin

**Affiliations:** Department of Materials Science and Engineering, National Taiwan University, No. 1, Sec. 4, Roosevelt Road, Taipei 10617, Taiwan; m.rizqiramadhan12@gmail.com (M.R.R.F.); f10527a06@ntu.edu.tw (H.-J.C.)

**Keywords:** cryogenic treatment, martensitic stainless steel, retained austenite, microstructure, mechanical properties

## Abstract

Martensitic stainless steel (MSS) is widely used in several parts of automobiles where high strength, hardness, and corrosion resistance are required. However, the metastability of retained austenite can transform into martensite under severe deformation, adversely affecting material properties. Cryogenic treatments (CTs) have been extensively employed in iron-based alloys for fastener application due to their advantageous effect. This study explores the heat treatment processes applied to 13Cr-2Ni-2Mo martensitic stainless steel (MSS), including austenitizing, cryogenic treatment, and tempering cycles. Cryogenic treatment at (−150 °C) for varying durations, followed by tempering at 200 °C for 2 h, and the impact of post-cryogenic tempering at 200 °C for different tempering duration on the microstructure and mechanical properties were evaluated. Experimental results indicate that the sample quenched at 1040 °C for 2 h (CHT) contains lath martensite, retained austenite, δ-ferrite, and undissolved carbide precipitation. Compared to as-quenched samples, hardness decreased by 5.04%, 7.24%, and 7.32% after tempering for 2 h, 5 h, and 10 h, respectively. Extending cryogenic durations to 2 h, 12 h, and 20 h promoted nucleation of a mixture of M_3_C and M_23_C_6_ small globular carbides (SGCs) and grain refinement but resulted in hardness reductions of 5.04%, 5.32%, and 8.36%, respectively. The reduction in hardness is primarily attributed to a decrease in solid solution strengthening and promoted carbide coarsening.

## 1. Introduction

Martensitic stainless steels (MSSs) are extensively used in automobiles, turbine blades, surgical instruments, bearings, and fasteners due to their excellent strength, hardness, wear resistance, and corrosion resistance [1]. A recent advancement in automotive manufacturing involves the cold forming of high-strength steel and martensitic stainless steel components, reducing material waste and improving mechanical performance. This process allows the production of complex geometries with enhanced strength and ductility, making it suitable for critical applications such as chassis reinforcements and crash-resistant structures. Main properties such as formability must be optimized, alongside maintaining sufficient corrosion resistance and fatigue durability for extended service life [2].

To fulfill these requirements, microstructure and mechanical properties need to be precisely managed. In MSSs, the presence of certain phases, such as retained austenite and delta ferrite, during heat treatment and quenching may have an adverse impact on the mechanical properties. During the annealing of certain martensitic stainless steels, reverted austenite can achieve partial stabilization and persist at room temperature. This stabilization primarily arises from chemical factors, such as the partitioning of austenite-stabilizing elements, including its lamellar morphology. Retained austenite (RA) is metastable at room temperature and can transform under heavy load or stress during component operation. This transformation can lead to dimensional changes, negatively impacting the durability of tools and components during operation. Thus, it is crucial to eliminate RA from most stainless steels before use to ensure optimal functional performance [3,4,5]. The impact of δ-ferrite on martensitic-based steel remains unclear; however, the consensus among researchers is that its presence may deteriorate the mechanical properties. Specifically, δ-ferrite can adversely influence fatigue fracture as cracks are prone to nucleate in the regions containing δ-ferrite [6].

Cryogenic treatment (CT) is widely recognized for its effectiveness in reducing the fraction of retained austenite and stabilizing phases at room temperature. During CT, the material is held at a temperature significantly lower than the martensite finish (M_f_) temperature for a specified duration, followed by a reheating process to room temperature. This treatment promotes the conversion of retained austenite into martensite and induces the formation of finely dispersed secondary carbide precipitates within nucleation sites [4,5,6,7,8,9,10,11].

This study focuses on the development of novel stainless steel 13Cr-2Ni-2Mo due to its superior mechanical properties compared to conventional martensitic stainless steel such as AISI 410 and AISI 420. This study will use martensitic stainless steel for experiments and design a series of heat treatments, including cryogenic treatments, to measure its hardness and observe microstructural differences. The objective is to understand the influence of cryogenic treatments and tempering duration towards the distribution of carbide and other phases.

## 2. Materials and Methods

### 2.1. Materials and Heat Treatments

In this study, experiments were conducted using a total of 7 samples of 13Cr-2Ni-2Mo martensitic stainless steel rods. The test rod used in this study was subjected to rod rolling and two annealing processes at above Ac1 temperature to relieve internal stresses, resulting in a final diameter of 3.5 mm for the rod used in this experiment. The composition of the 13Cr-2Ni-2Mo martensitic stainless steel rod was confirmed using a Spark Optical Emission Spectrometer (Spark OES) with four different points with the average elemental analysis results presented in Table 1.

**Figure 1:**
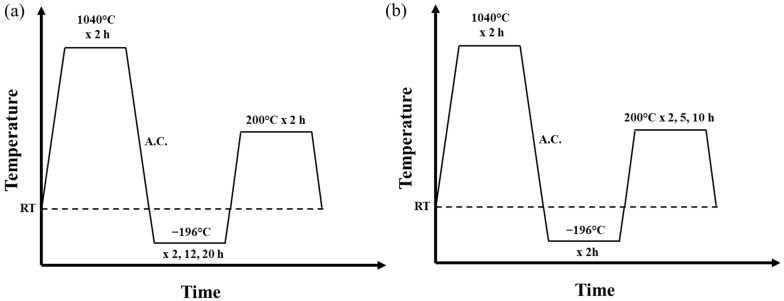
Heat treatment process for (**a**) different cryogenic duration; (**b**) different tempering duration (note: cryogenic treatment in liquid nitrogen was performed immediately after air-cooling (A.C.) quenching, followed immediately by tempering).

For simplicity, this paper designates conventional heat treatment as CHT, which excludes cryogenic treatment, cryogenic treatment as CT, and tempering as T. The as-received material underwent solution treatment at 1040 °C for 2 h, followed by air-cooling as a conventional heat treatment (CHT) sample. The as-quenched sample was subsequently tempered at 200 °C for 2 h (CHT/T2) immediately. Both samples served as reference conditions for comparison against other experimental groups. To evaluate the effect of tempering duration on cryogenically treated CHT samples, various tempering times of 2 h, 5 h, and 10 h were applied after immersing the CHT sample in liquid nitrogen at −196 °C immediately after quenching. In addition, to study the influence of cryogenic treatment duration, samples were subjected to cryogenic treatment for 2, 12, and 20 h after CHT, followed by tempering at 200 °C for 2 h. The experimental design is illustrated in Figure 1 and Table 2.

### 2.2. Microstructure and Mechanical Properties Analysis

Samples were etched on glyceregia solution (3 parts of HCl, 2 parts of glycerol and 1 part of HNO_3_) for 1 min. A Leica DM2500M optical microscope (Leica Microsystems, Wetzlar, Germany) and a Canon EOD 5D MARK II camera (Future-Tech, Tokyo, Japan) were used for this experiment. To investigate microscale secondary phase particles, secondary electron images were captured at 20 kV accelerating voltage using a FEI NOVA scanning electron microscope. In this experiment, the FEI Tecnai G2 F20 200 kV FEG-TEM (Thermo Fisher Scientific, Hillsboro, OR, USA) was used for microstructure analysis. Bright-field imaging and diffraction patterns were employed to observe the morphology and analyze the crystal structure of martensite, austenite, and precipitates. The incorporated Energy-Dispersive Spectrometer (EDS) with an X-MAX 80 detector facilitated chemical composition analysis to observe the element of the precipitate. X-ray diffraction measurements were conducted using a D2 PHASER X-ray Powder Diffractometer (Bruker Corporation, Billerica, MA, USA). The XRD analysis utilized Cu-Kα radiation with a wavelength of 1.54060 Å with a 300 W high-voltage power supply. A JeoL JSM-7800F Prime field emission gun scanning electron microscope equipped (JEOL Ltd, Tokyo, Japan) with a NordlysNano EBSD detector (Oxford Instruments, Wycombe, UK) with a working voltage of 20 kV was used to receive backscattered electrons was used for the measurement of material crystal orientations, providing information such as microstructural composition, texture, orientation differences, phase proportions, grain size, and morphology.

XRD results were used to investigate the proportions of retained austenite and martensite. Proportional retained austenite was calculated using the following equation:

(1) $$V_{\gamma }=\frac{1-V_{C}}{1+\left(\frac{R_{\gamma }^{hkl}}{R_{\propto \prime }^{hkl}}\right)\left(\frac{I_{\propto \prime }^{hkl}}{I_{\gamma }^{hkl}}\right)}$$

where V_γ_ is the volume percent of retained austenite, V_C_ is the volume percent of carbide, which were calculated from SEM microstructure using ImageJ software (version 1.54p, National Institutes of Health (NIH), Bethesda, MD, USA), and Iα′ and Iγ are the integrated intensities measured for a single preselected martensite and austenite peak line, respectively. R_α′_ and R_γ_ are theoretical intensity values for the same hkl planes [12,13]. Six diffraction peaks of (211)α′, (200)α′, (110)α′, (111)γ, (200)γ, and (220)γ were selected to measure the integrated intensity.

After applying conventional heat treatment and cryogenic treatment, hardness measurements were performed using an FM-810 Vickers hardness tester (Future-Tech, Tokyo, Japan) at a load of 1000 g and a dwell time of 10 s. Fifteen randomly selected points were tested on the material surface and the average hardness value. In this experiment, the MTS 810 universal testing machine (MTS Systems Corporation, Eden Prairie, MN, USA) was employed to conduct the three-point bend test at a downward speed of 0.48 mm/min. The three-point bending test was chosen over the tensile test to evaluate plastic deformation in martensitic stainless steel with minimal sample preparation. The testing method followed ASTM E290-22 [14] with a span of 15 mm and a sample length of 30 mm, which was calculated using the standard span formula as in the equation below:

(2) $$C=\left(2r+3t\right)\pm \frac{t}{2}$$

with C as the distance between lower supports, r as the radius of the pin end, t as the specimen thickness, d as the round specimen diameter, and was the specimen width [14]. The indenter and sample rod diameters are 4.90 mm and 3.50 mm, respectively. The applied force starts at zero and fracture marks the endpoint. The resulting force–displacement graph is used to assess the material tensile strength and ductility.

## 3. Results and Discussions

### 3.1. Microstructure of As-Quenched and Conventionally Quenched-Tempered Samples

**Figure 2:**
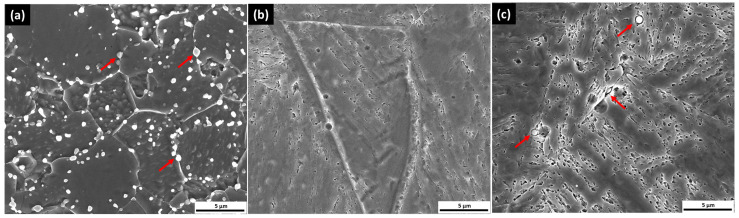
SEM microstructure of sample: (**a**) as-received sample; (**b**) CHT; (**c**) CHT/T2 (red arrow: carbide precipitation).

The as-received microstructure contains distributed spherical carbide inside the grain and along the grain boundary, as shown in Figure 2a. The majority of carbides were redissolved following austenitization at 1040 °C for 2 h as illustrated in Figure 2b, indicating that this treatment parameter effectively facilitates the dissolution of a substantial quantity of alloying elements. Precipitation initiates along the grain boundaries after 2 h of tempering, as shown in Figure 2c, as these regions offer accelerated pathways for atomic diffusion compared to the inside grain due to their higher atomic disorder. Consequently, grain boundaries serve as favorable sites for carbon atom migration during the tempering process [4,15].

**Figure 3:**
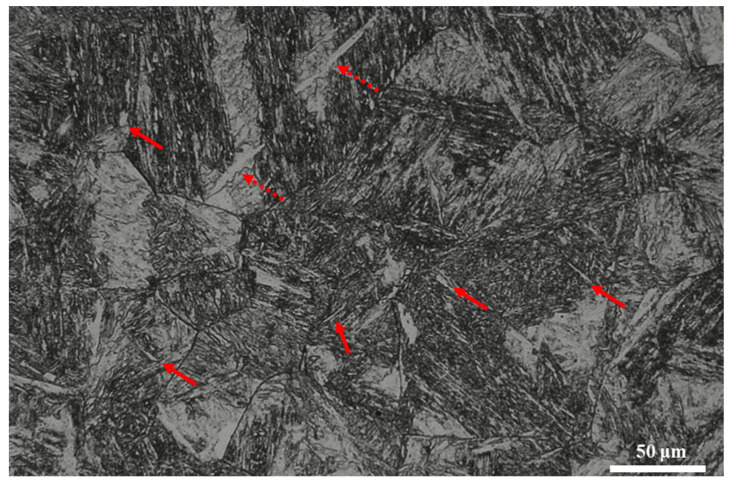
Optical micrographs of sample CHT (dashed red arrow: δ-Fe as a platelet-like structure; solid red arrow: δ-Fe as isolated islands).

Optical microscope examination reveals that low amounts of δ-ferrite phase exist in sample CHT, which consists of a small discontinuous platelet-like shape with a size of around 1–50 μm, as shown in Figure 3 (red arrow). The cause is that ferrite-stabilizing elements like Cr and Mo diffuse and concentrate at elevated temperatures, leading to an increase in the Cr equivalent (Cr_eq_). This promotes the formation of delta ferrite more readily when austenitized at higher temperatures. As is well known, δ-ferrite is a relatively soft phase within martensite, which consequently decreases the overall strength of the steel [3,15].

The low tempering temperature, typically below 250 °C inhibits the widespread precipitation of carbides throughout the metal matrix and minimizes the formation of Cr-rich carbides in martensitic stainless steels. This material is designed for enhanced wear and corrosion resistance, which is crucial to carefully managing carbide formation, as an excessive presence of carbides could adversely affect the alloy’s corrosion resistance. Moreover, tempering at elevated temperatures ranging from 480 to 600 °C may result in reduced corrosion resistance owing to the onset of sensitization and reversed austenite formation [1,15,16,17].

**Figure 4:**
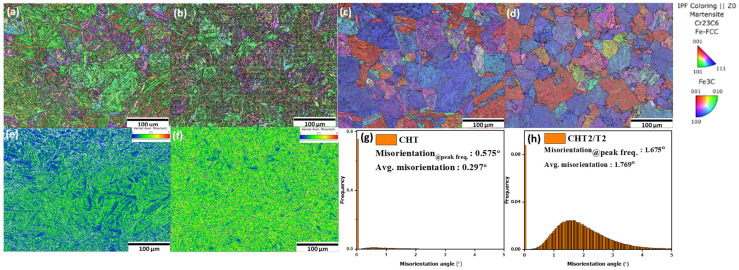
EBSD analysis IPF-Z images of (**a**) CHT and (**b**) CHT/T2; parent austenite grain boundary (PAGB) of (**c**) CHT and (**d**) CHT/T2; KAM maps of (**e**) CHT and (**f**) CHT/T2; average misorientation histogram of (**g**) CHT and (**h**) CHT/T2.

During tempering, carbide precipitates begin to occur within the martensite matrix. These carbides tend to form at dislocations, grain boundaries, and interfaces within the martensitic microstructure. The precipitation of carbides may assist in refining the grain boundaries and substructures; thus, the grain size appears smaller in the tempered condition. The average prior austenite grain boundary (PAGB) size decreased to 42.54 ± 12.69 μm following conventional tempering, as illustrated in Figure 4d, compared to the as-quenched sample, which exhibited a PAGB size of 51.15 ± 10.17 μm (Figure 4c). KAM maps on both samples were observed to evaluate the degree of misorientation. In this paper, the threshold for general grain boundary misorientation is set at 5°. This implies that misorientations greater than 5° were excluded from the calculation of local misorientation, as they are attributed to grain boundaries rather than the accumulation of geometrically necessary dislocations (GNDs). The GND density can be extrapolated using the formula provided below [4,16,18]:

(3) $$\rho^{GND}=\frac{2\theta }{\mu b}$$

ρ^GND^ represents the geometrically necessary dislocation (GND) density at each point, θ denotes the local misorientation, μ refers to the unit length (which is 100 nm in this paper), and b is the Burgers vector. The CHT/T2 sample has a relatively higher average misorientation degree compared to CHT. The increase in misorientation degree during tempering can be attributed to a combination of transformation from retained austenite to secondary martensite while simultaneously carbon diffuses during tempering from martensite to another RA site which may introduce local changes in orientation, contributing to higher misorientation degrees. Therefore, according to Equation (3), the CHT/T2 sample exhibits a higher dislocation density compared to the as-quenched sample which contrasts with findings from other authors that indicate lower dislocation densities when tempering occurs at elevated temperatures (above 500 °C) due to stress relieve occurs simultaneously [16,17,18].

In this study, the relatively lower content of austenite-stabilizing elements, such as Ni, may result in an even lower M_s_ temperature. Additionally, fine carbides precipitate within the martensitic matrix and along grain boundaries during tempering. These precipitates can generate localized stress concentrations, increasing grain boundary misorientation [18,19,20,21,22,23,24,25]. Consequently, tempering at such a low temperature does not necessarily result in a lower misorientation degree compared to tempering at elevated temperatures [16].

### 3.2. Effects of Cryogenic Treatment

#### 3.2.1. Microstructural Characterization

**Figure 5:**
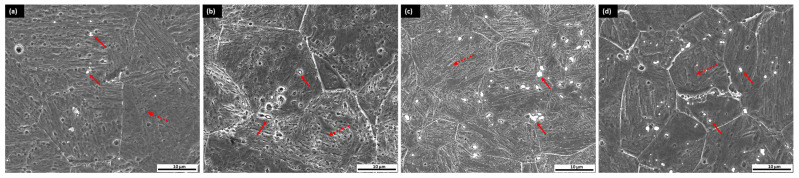
FESEM microstructure of sample: (**a**) CHT/T2 without cryogenic treatment; (**b**) CT2/T2; (**c**) CT12/T2; (**d**) CT20/T2 (solid red arrow: carbide; dashed red arrow: tempered martensite).

As illustrated in Figure 5, small globular carbides precipitated in the CHT sample after tempering and in samples subjected to cryogenic treatment, particularly along the grain boundaries. The carbides predominantly nucleate along the boundaries of martensitic laths and exhibit nanocluster growth within carbon-enriched regions across all samples, which is consistent with findings reported by other researchers [5,6,9]. As depicted in Figure 4a, the sample without cryogenic treatment exhibits the lowest carbide distribution.

**Figure 6:**
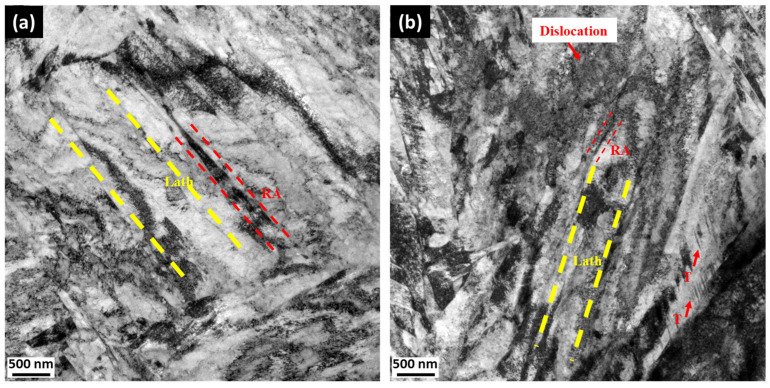
TEM micrographs of sample: (**a**) CHT/T2 without cryogenic treatment; (**b**) with cryogenic treatment CT2/T2 (T: twin martensite).

Figure 6 shows the TEM morphologies of CHT/T2, which contain lath martensite matrix and retained austenite, while after cryogenic treatment CT2/T2, crystal defects such as twin martensite were observed. It shows that the size of martensite lath decreases significantly with the cryogenic treatment with an average width of approximately 417 nm (Figure 6b), while the conventional QT process exhibits a larger average lath martensite with a width of approximately 524 nm (Figure 6a) [19]. TEM analysis reveals that cryogenic treatment induces a higher dislocation density within the matrix, primarily attributed to the transformation of retained austenite into fresh martensite [26,27,28,29,30,31,32,33,34,35,36,37].

**Figure 7:**
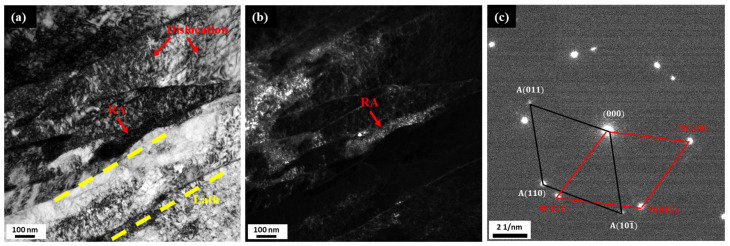
TEM micrograph of sample CT2/T2 with cryogenic treatment: (**a**) bright field image; (**b**) dark field image; (**c**) SAED pattern of retained austenite (RA).

In addition, a smaller retained austenite width is observed in Figure 7 in the form of thin films with an approximate width of 100 nm located between martensite laths. Based on the identification of diffraction spots, these black elongated substances are retained austenite, which aligns with the K-S relationship with martensite, i.e., ${<011>}_{\gamma -Fe}∥{<111>}_{\alpha -Fe}$ and ${\left\{111\right\}}_{\gamma -Fe}∥{\left\{011\right\}}_{\alpha -Fe}$. These retained austenite structures impede dislocation movement caused by external factors, thereby contributing to the improvement of hardness [8,23]. At cryogenic temperatures, C atoms diffuse from martensite to martensite/austenite interfaces due to lattice strain energy, hence enriching the retained austenite with C atoms. This C enrichment stabilizes the retained austenite during recovery to ambient temperature which prevents its transformation to martensite. This process is known as the thermal stabilization of austenite [3].

**Figure 8:**
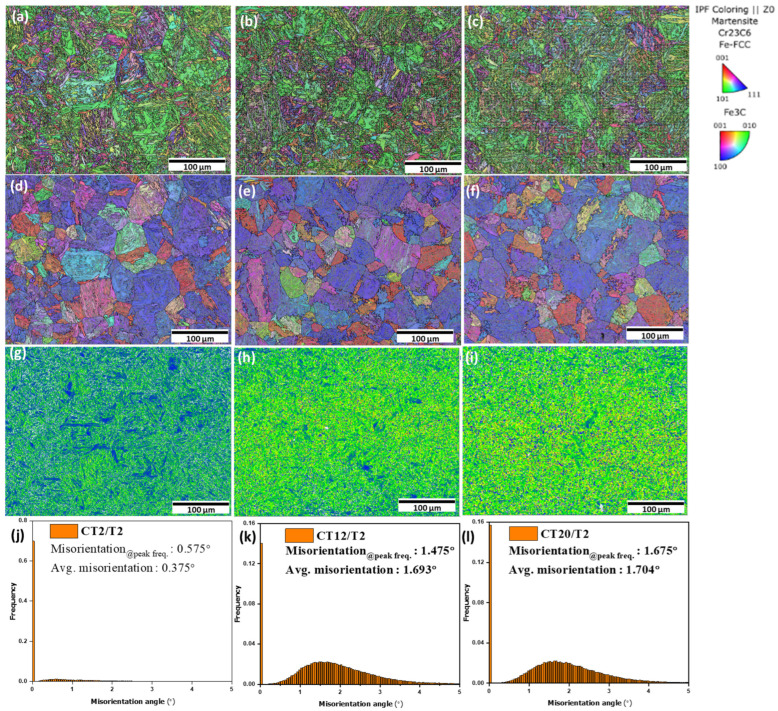
EBSD analysis IPF-Z images of (**a**) CT2/T2, (**b**) CT12/T2, and (**c**) CT20/T2; parent austenite grain boundary (PAGB) of (**d**) CT2/T2, (**e**) CT12/T2, and (**f**) CT20/T2; KAM maps of (**g**) CT2/T2, (**h**) CT12/T2, and (**i**) CT20/T2; and average misorientation histogram of (**j**) CT2/T2, (**k**) CT12/T2, and (**l**) CT20/T2.

The influence of cryogenic treatment duration on the microstructure is depicted in Figure 8. As the treatment duration increases, the size of the lath martensite decreases, as shown in Figure 8a–c, and the prior austenite grain boundaries (PAGB) also become smaller, as shown in Figure 8d–f from 41.14 ± 9.22 μm, 27.58 ± 9.06 μm, and 24.84 ± 8.05 μm for sample CT2/T2, CT12/T2, and CT20/T2, respectively. This phenomenon can be attributed to two main factors. First, the transformation of retained austenite to martensite is enhanced with longer treatment durations, resulting in a refined martensitic structure. The newly formed martensite typically exhibits smaller sub-grains, which contributes to an overall reduction in grain size. Continuous cooling to cryogenic temperatures enhances martensite transformation energy and instability [3]. Second, extended cryogenic treatment promotes finer carbide precipitation along the grain boundaries. These precipitates can act as pinning forces at the grain boundaries, thereby restricting grain growth. The presence of carbides stabilizes the microstructure and inhibits coarsening of the grains [4,5,7,8,9,10,11].

The austenite-to-martensite transformation induces a positive volumetric effect, with the magnitude of this volumetric change increasing alongside the carbon content, which is consistently accompanied by plastic deformation in the newly formed martensite. Recent research has demonstrated that the martensitic transformation of retained austenite at low temperatures leads to plastic deformation of the newly formed martensite [3,4,5]. This deformation results in immobile carbon atoms being trapped by dislocations, forming carbon clusters that act as nucleation sites for finer carbide particles during tempering, thereby affecting the carbide distribution [5,9]. M. Villa et al. [32] studied the development of compressive strains in austenite following its transformation into martensite at low temperatures. This explains the observation of higher average misorientation degrees in the KAM results as the cryogenic treatment duration is extended [5]. Figure 8g,h,j show the local kernel average misorientation (KAM), which represents the strain concentration results for low angle boundary, and high KAM indicates a high-strain region. Following cryogenic treatment, the distribution of strain zones became more uniform. Additionally, regions of high strain were predominantly localized at the interfaces between the martensite and RA phases [4]. The KAM values of the martensite and RA phases increased after the cryogenic treatment from 0.375°, 1.693°, and 1.704° of sample CT2/T2, CT12/T2, and CT20/T2, respectively.

**Figure 9:**
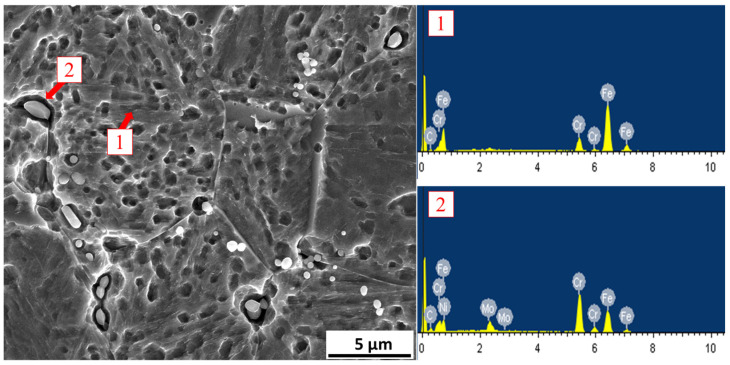
EDS spectra of sample after cryogenic for 20 h and tempering for 2 h (CT20/T2). Point 1: matrix; point 2: Mo-rich carbide.

The findings, consistent with S. Li et al. [3], reveal a substantial number of carbides precipitated from the martensitic matrix, with the carbides being relatively uniformly distributed after a 20 h cryogenic treatment. Figure 9 illustrates the EDS spectra, where nanoscale and microscale small globular carbides (SGCs) enriched with Mo were identified. The presence of these carbides is a typical outcome observed following cryogenic treatment [5,36,37,38,39].

**Figure 10:**
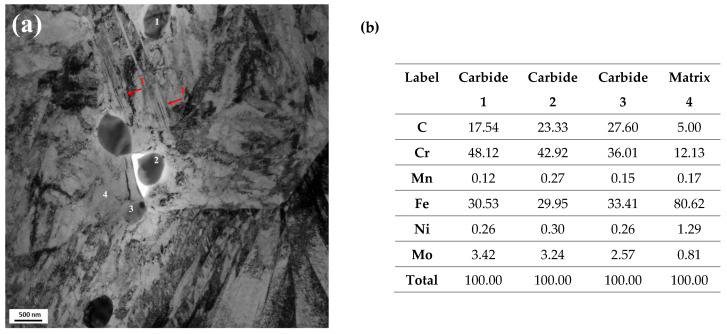
(**a**) TEM micrograph of CT2/T2; (**b**) TEM-EDS point label (T: twin martensite).

**Figure 11:**
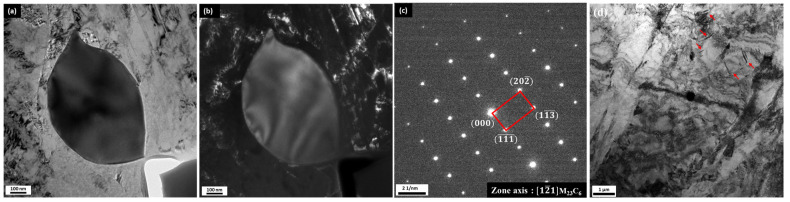
TEM image results after CT at −150 °C and tempering at 200 °C (CT2/T2): (**a**) bright field image; (**b**) dark field image; (**c**) SAED for M_23_C_6_; (**d**) TEM micrograph shows few instances of needle-shape M_3_C (red arrow).

To examine the microstructure following cryogenic treatment, TEM analysis was performed on the CT2/T2 sample. Figure 10a shows the morphology and distribution of retained austenite and lath martensite, which include a small globular carbide. At lower tempering temperatures, the diffusion rate of chromium atoms remains slow, which leads to the preferential formation of a few instances of Fe-rich M_3_C (Figure 11d). However, since cryogenic treatment was applied prior to tempering, it facilitated nanoscale carbide nucleation within the matrix and followed by carbide stabilization after tempering [25,40,41,42,43,44,45]. As depicted in Figure 11, the carbides observed were predominantly a mixture of M_23_C_6_ with a size of approximately 500 nm and M_3_C. M_23_C_6_ SGC types were observed to be enriched with Cr and Mo, as shown in Figure 10b; this carbide most likely originated from undissolved carbide during austenitizing. The cryogenic treatment enhances the formation and stabilization of this nanoscale globular carbide M_23_C_6_ through diffusion as a reaction below [25,44,45]:

Matrix → M_3_C → M_7_C_3_ → M_23_C_6_ (4)

#### 3.2.2. Mechanical Properties

**Figure 12:**
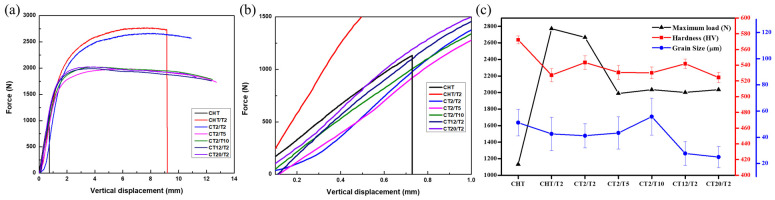
(**a**) Three-point bending test results at different heat treatments; (**b**) larger picture for (**a**); (**c**) maximum load, hardness value, and grain size all samples.

As in Figure 12c, sample CHT exhibits the highest hardness among all samples (572 ± 4.99 HV). The primary strengthening mechanisms in tempered martensitic steel are influenced by fine grain strengthening, solid solution strengthening, dislocation strengthening, and precipitation strengthening. In the as-quenched sample, the highest hardness value can be attributed primarily to solid solution strengthening. Soluble atoms cause distortion in the Fe crystal lattice, creating a stress field that hinders dislocation movement due to the interaction between the stress field and the dislocations. Furthermore, since the as-quenched sample lacks carbide precipitation, precipitation strengthening plays a negligible role in its hardness and strength. Based on the KAM results, the misorientation degree, which correlates with dislocation density (and thus dislocation strengthening) also appears to have little effect on hardness [4,17,40]. The sample subjected to 2 h tempering (CHT/T2) demonstrates the lowest hardness, which is 527 ± 8.27 HV. This reduction in hardness can be attributed to the diffusion of alloying elements such as C, Cr, and Mo from the solid solution and carbide coarsening. These carbides precipitate immediately due to the higher fraction of retained austenite in conventional quenching and tempering (QT) treatments, facilitating more rapid carbon diffusion in the FCC phase. This process reduces solid solution strengthening and, as a result, causes a decrease in the overall hardness [16,41,42,43,44,45].

**Figure 13:**
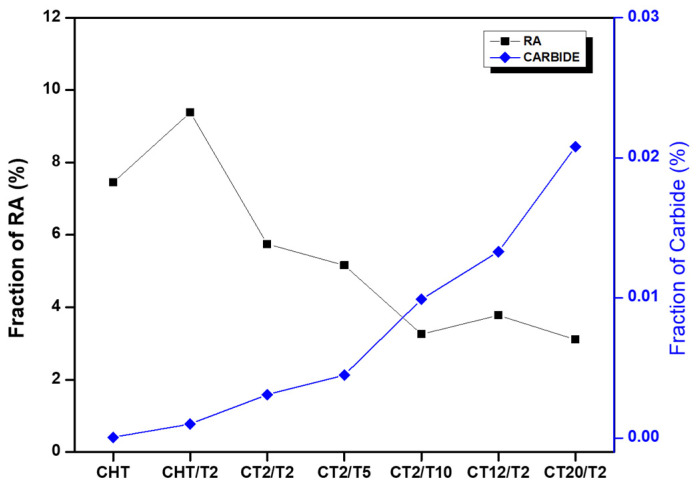
Fraction of RA and carbide.

Cryogenic treatment before tempering was applied exhibited a higher hardness value of 543 ± 8.67 HV for the CT2/T2 sample, as observed in Figure 12c. Although there is a slight decrease in hardness for sample CT12/T2, this reduction was not significant. The combined effects of higher dislocation density, as illustrated in Figure 8l, precipitation strengthening, and a lower fraction of retained austenite, as shown in Figure 13, contribute substantially to the hardness improvement compared to conventional QT treatment. Conversely, in the sample subjected to 20 h of cryogenic treatment, the hardness deteriorates markedly due to the substantial amount of solute migrating out of the matrix to form carbides. Additionally, the chromium solute present in the martensite matrix and δ-ferrite may be rejected into the matrix, leading to the formation and coarsening of carbides at the interface between the δ-ferrite and the martensitic phase, as illustrated in Figure 9. This was supported by the rightward shift of the martensite peak on the XRD result observed in Figure 13 [7,8,9,26,36].

Figure 12a shows the mechanical properties of all samples from the three-point bend test. Surprisingly, the sample without cryogenic treatment (CHT/T2) has the highest maximum load, which is 2772 N compared with all samples. On the other hand, CHT reached only 1133 N before fracturing, primarily due to its brittleness. This phenomenon is attributed to carbon atoms being trapped within the martensitic structure, causing lattice distortion and significantly increasing brittleness. The material’s limited capacity for plastic deformation makes it highly prone to cracking under applied stress. The yield strength increments in stainless steels can be expressed using the following equation below:

(5) $$\sigma_{y}={∆\sigma }_{0}+{∆\sigma }_{s}+\sqrt{{∆\sigma }_{p}^{2}+{∆\sigma }_{\rho }^{2}}+k_{y}d^{-1/2}$$

In this equation, Δ*σ*_0_ represents the friction stress for steel, often referred to as the Peierls–Nabarro force; Δ*σ*_*s*_ accounts for solid solution hardening; Δ*σ*_*ρ*_ is associated with the strengthening effect of dislocations within the martensitic laths, and Δ*σ_p_* corresponds to precipitation hardening. The term *k*_*y*_*d*^−1/2^ describes the grain boundary strengthening, where *k_y_* is the Hall-Petch slope and d represents the effective grain size or the spacing of high-angle grain boundaries [25,40,46].

The CHT/T2 sample exhibited a relatively higher maximum load of 2772 N with the lowest vertical displacement at 9.1 mm, and fracture occurs during applied deformation compared to all cryogenic treatment samples, which demonstrated relatively better toughness without fracture with loads ranging from 1989 N (CT2/T5) to 2035 N (CT20/T2). Specifically, the maximum loads for the cryogenic treatment samples were recorded as follows: 2665 N (CT2/T2), 1989 N (CT2/T5), 2034 N (CT2/T10), 2001 N (CT12/T2) and 2035 N (CT20/T2). The primary reason stems from the tempering conditions referred to in Equation (5), which promote greater carbide precipitation along grain boundaries and induce more pronounced lattice distortion from dislocation accumulation during martensitic transformation and carbide formation, this process also stabilizes the retained austenite. The CHT/T2 sample, with its higher retained austenite content, demonstrates improved toughness despite lower hardness. This behavior can be attributed to the reduced solid solution strengthening effect, as confirmed by XRD results, which allows the sample to sustain a higher maximum load before fracture even with its lower hardness [47,48,49,50,51,52,53].

It is widely recognized that the aging or tempering of as-hardened steel can stabilize retained austenite (R_A_). During the aging or tempering process, carbon atoms initially diffuse from the martensite (M) phase to the M/RA interfaces, where they segregate and anchor the typically mobile dislocations. In the later stages, the carbon atoms begin to diffuse into the austenite phase, leading to an increase in the carbon content within the austenite [47,48,49,50,51].

In terms of quantifying residual austenite, analyses were conducted on the austenitized specimens using XRD, as shown in Figure 13, retained austenite (RA) volume fraction revealed a decreasing trend after cryogenic treatment, reducing from 9.53% in the CHT sample to 8.43% after 2 h of immersion (CT2/T2) and further to 6.51% after 20 h of immersion (CT20/T2). This downward trend is consistent with the findings of Li S. et al. [3] for stainless steel and Kang C. et al. [4] for steel in their respective experiments, which conclude that cryogenic treatment is an effective way to reduce retained austenite.

#### 3.2.3. Microstructural Evolution with Different Tempering Duration

Cryogenic treatment is an effective way to reduce retained austenite to an acceptable level. In addition to the retained austenite reduction, a significant increased amount of additional small globular carbides in the microstructure of the cryogenically treated material [7,8,9,10,11].

**Figure 14:**
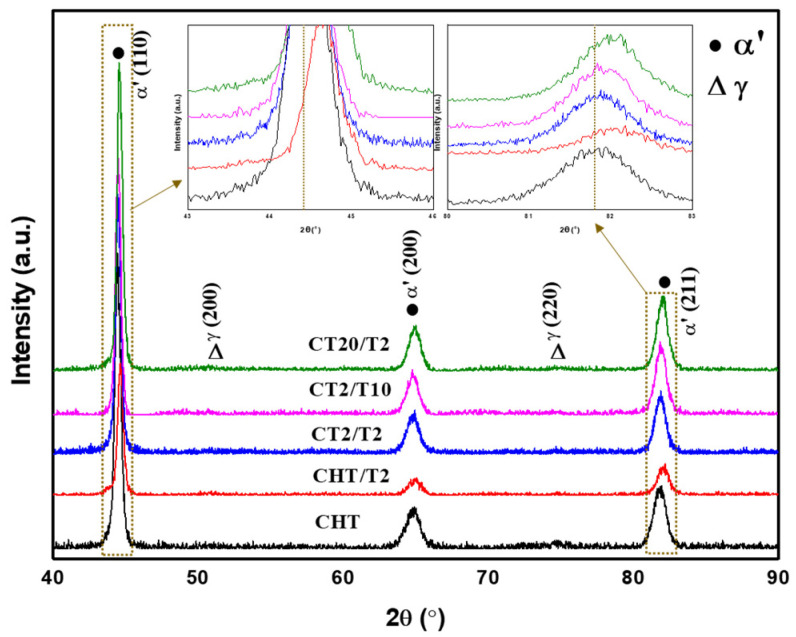
XRD spectra for CHT and CT specimens.

A longer tempering duration exhibits a decreasing trend in Cr content as the tempering time increases, as reported by Jiang W. et al. [1]. This phenomenon can be attributed to carbide precipitation which is evident from the SEM analyses. XRD result Figure 14 illustrates the martensite peak is the predominant peak, with a smaller intensity peak corresponding to retained austenite clearly visible. However, distinguishing between the martensite and body-centered cubic (bcc) δ-ferrite peaks is challenging due to their peak overlap. The martensite peak for sample CHT/T2 displays a significant rightward shift compared to sample CHT, which indicates that conventional QT treatment develops Cr-depleted regions and a reduction in the martensite lattice parameter. This shift results from the depletion of C and Cr in the martensite matrix as it forms a solid solution [1,17,23].

**Figure 15:**
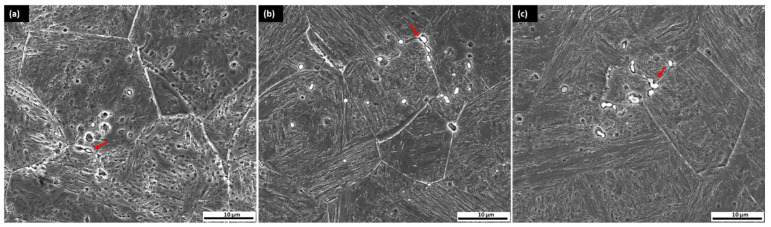
FESEM microstructure of sample: (**a**) with cryogenic treatment CT/T2; (**b**) CT2/T5; (**c**) CT2/T10.

RA content was reduced for the sample after 10 h tempering, which was 6.04% compared to 2 h tempering at 8.43%. The main reasons were as follows: first, carbon diffusion during prolonged tempering, carbon atoms diffuse more significantly from the retained austenite into the martensitic matrix and decrease the stability of the austenite. This depletion of carbon facilitates the transformation of austenite into martensite. Second, carbide precipitation and extended tempering increase the precipitation of carbides, particularly M_23_C_6_, which further depletes carbon from the austenite, reducing its stability and encouraging the transformation of austenite into martensite [17,47,48,49,50,51]. As shown in Figure 15, an increased amount of carbide (red arrow) precipitation was evident, particularly in sample CT2/T10, which likely contains Mo- and Cr-rich carbides within the martensitic matrix.

**Figure 16:**
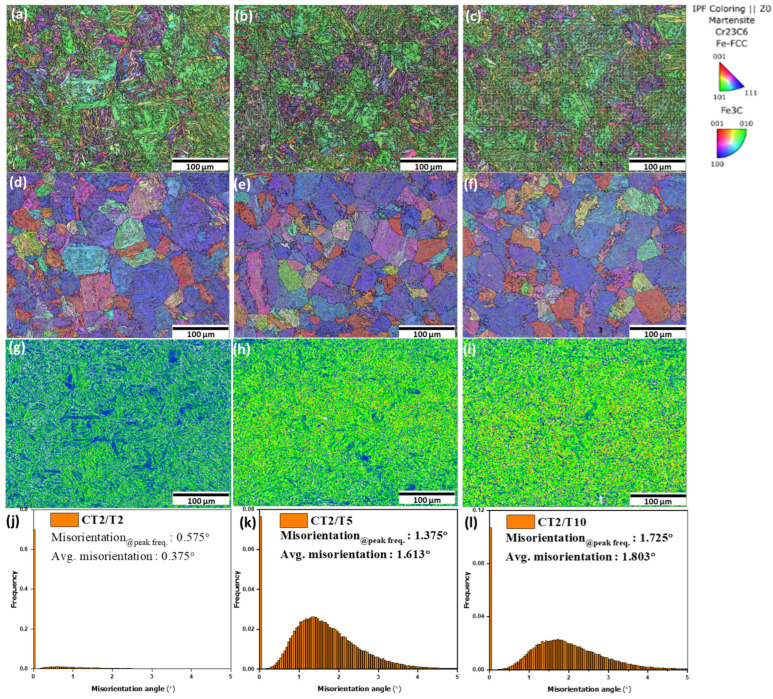
EBSD analysis IPF-Z images of (**a**) CT2/T2, (**b**) CT2/T5, and (**c**) CT2/T10; parent grain boundary (PAGB) of (**d**) CT2/T2, (**e**) CT2/T5, and (**f**) CT2/T10; KAM maps of (**g**) CT2/T2, (**h**) CT2/T5, and (**i**) CT2/T10; average misorientation histogram of (**j**) CT2/T2, (**k**) CT2/T5, and (**l**) CT2/T10.

The hardness decreases with extended tempering durations from 543 ± 8.67 HV, 531 ± 8.73 HV, and 530 ± 7.49 HV for samples CT2/T2, CT2/T5, and CT2/T10, respectively. The hardness observed after tempering was influenced not only by the dissolution of carbon atoms but also by the recovery of the martensitic structure at a low tempering temperature of 200 °C. As tempering progresses, the size and quantity of carbides tend to increase [42,43,44,45]. Larger PAGB were observed as well as extended tempering duration refer to Figure 16d–f from 41.14 ± 9.22 μm, 43.24 ± 12.24 μm, and 55.73 ± 14.08 μm for CT2/T2, CT2/T5, and CT2/T10, respectively. A longer tempering duration can lead to a decrease in hardness. This was attributed to the generation of non-uniformly distributed coarser particles, making them less effective in acting as pinning points, thus reducing the overall hardness [5].

## 4. Conclusions

In this study, it was observed that both cryogenic treatment and tempering duration influence microstructure and mechanical properties with the following results:

1. The as-quenched sample (CHT) of 13Cr-2Ni-2Mo contains lath martensite, retained austenite, undissolved carbide, and δ-ferrite. Cryogenic treatment (CT) facilitates the conversion of retained austenite into martensite, resulting in increased hardness compared to conventional QT treatment (CHT/T2).
2. The predominant carbide types are nanosized M_23_C_6_ and a few instances of M_3_C (cementite), along with a smaller grain size distribution relative to the CHT process. Following cryogenic treatment durations of 2 h, 12 h, and 20 h, the average prior austenite grain boundary (PAGB) sizes were found to be 41.14 ± 9.22 μm, 27.58 ± 9.06 μm, and 24.84 ± 8.05 μm, respectively.
3. Variations in tempering duration can lead to an increase in PAGB size, which is associated with the coarsening of carbides and grain size. After tempering for 2 h, 5 h, and 10 h at 200 °C, the PAGB sizes were 41.14 ± 9.22 μm, 43.24 ± 12.24 μm, and 55.73 ± 14.08 μm, respectively.
4. Extended durations of cryogenic treatment can result in decreased hardness compared to the as-quenched sample, primarily due to a reduction in the solid solution strengthening effect. After cryogenic treatment for 2 h, 12 h, and 20 h followed by tempering, the hardness values relative to the as-quenched state were 543 ± 8.67 HV (−5.04%), 541 ± 6.12 HV (−5.32%), and 524 ± 6.53 HV (−8.36%), respectively.
5. The hardness of samples subjected to different tempering durations post-cryogenic treatment were 2 h, 5 h, and 10 h at 200 °C, compared to the as-quenched sample, yielded values of 543 ± 8.67 HV (−5.04%), 531 ± 8.73 HV (−7.24%), and 530 HV ± 7.49 (−7.32%), respectively.
6. 13Cr-2Ni-2Mo martensitic stainless steel, which is intended for automobile applications requiring high hardness and good toughness, and a combination of cryogenic treatment for 2 h at −150 °C, followed by tempering for 2 h at 200 °C (CT2/T2), was the optimal processing route in this study. Cryogenic treatment contributes to a reduction in retained austenite content effectively, which prevents dimensional changes caused by austenite transformation during service, thereby avoiding failure and effectively improving the service life of the experimental steel.

## Figures and Tables

**Table 1:** Chemical composition of 13Cr-2Ni-2Mo in wt%.

MSS	C	Cr	Ni	Mo	Si	Mn	N	Fe
13Cr-2Ni-2Mo	0.17 ± 0.03	12.9 ± 0.05	1.8 ± 0.07	1.92 ± 0.06	0.36 ± 0.02	0.08 ± 0.01	0.12 ± 0.04	Bal.

**Table 2:** Specimen identification.

Identification	Description
As-received	Prior heat treatment
CHT	Conventionally heat-treated (as-quenched)
CHT/T2	As-quenched + Tempering at 200 °C for 2 h
CT2/T2	As-quenched + cryogenically at −150 °C for 2 h + Tempering at 200 °C for 2 h
CT2/T5	As-quenched + cryogenically at −150 °C for 2 h + Tempering at 200 °C for 5 h
CT2/T10	As-quenched + cryogenically at −150 °C for 2 h + Tempering at 200 °C for 10 h
CT12/T2	As-quenched + cryogenically at −150 °C for 12 h + Tempering at 200 °C for 2 h
CT20/T2	As-quenched + cryogenically at −150 °C for 20 h + Tempering at 200 °C for 2 h

## Data Availability

The original contributions presented in the study are included in the article, further inquiries can be directed to the corresponding author.
